# NOx Storage on BaTi_0.8_Cu_0.2_O_3_ Perovskite Catalysts: Addressing a Feasible Mechanism

**DOI:** 10.3390/nano11082133

**Published:** 2021-08-21

**Authors:** Vicente Albaladejo-Fuentes, María-Salvadora Sánchez-Adsuar, James A. Anderson, María-José Illán-Gómez

**Affiliations:** 1 Carbon Materials and Environment Research Group, Department of Inorganic Chemistry, Faculty of Science, Universidad de Alicante San Vicente del Raspeig, 03690 Alicante, Spain; vicentealbaladejo@gmail.com (V.A.-F.); dori@ua.es (M.-S.S.-A.); 2 Surface Chemistry and Catalysis Group, School of Engineering, University of Aberdeen, Aberdeen AB24 3UE, UK; j.anderson@abdn.ac.uk

**Keywords:** NOx storage mechanism, in situ DRIFTS, nitrites, nitrates, barium-titanium perovskite

## Abstract

The NOx storage mechanism on BaTi_0.8_Cu_0.2_O_3_ catalyst were studied using different techniques. The results obtained by XRD, ATR, TGA and XPS under NOx storage–regeneration conditions revealed that BaO generated on the catalyst by decomposition of Ba_2_TiO_4_ plays a key role in the NOx storage process. In situ DRIFTS experiments under NO/O_2_ and NO/N_2_ show that nitrites and nitrates are formed on the perovskite during the NOx storage process. Thus, it seems that, as for model NSR catalysts, the NOx storage on BaTi_0.8_Cu_0.2_O_3_ catalyst takes place by both “nitrite” and “nitrate” routes, with the main pathway being highly dependent on the temperature and the time on stream: (i) at T < 350 °C, NO adsorption leads to nitrites formation on the catalyst and (ii) at T > 350 °C, the catalyst activity for NO oxidation promotes NO_2_ generation and the nitrate formation.

## 1. Introduction

NOx Storage and Reduction (NSR) is one of the proposed technologies for the effective abatement of NOx from exhaust gas emitted by lean burn engines. A typical NSR catalyst is composed of a noble metal (mainly Pt and/or Rh) and an alkaline/alkaline earth oxide, both supported on high-surface aluminum oxide [1]. Based on the high chemisorption capacity of the alkaline/alkaline earth oxide, the NSR catalyst is able to store a high amount of NOx during the short time of the lean conditions step (oxygen rich atmosphere). Periodically, a reducing agent is fed in the gas exhaust system (rich conditions), which induces the release and reduction of the previously stored NOx and, as a result, the regeneration of the catalyst surface [2,3]. Thus, the NSR process involves several steps: (i) during the lean cycle, NO (main nitrogen oxide compound in gas exhaust conditions) to NO_2_ oxidation followed by its storage in the form of nitrites/nitrates on the surface of the basic oxide component of the catalyst, while afterwards, (ii) during the rich cycle, reductant feed or generation causes NOx release from the catalyst and their subsequent reduction to N_2_ [4].

A wide number of reports dealing with the study of the NOx storage and reduction mechanism on NSR model catalysts have been published [5,6,7,8,9,10,11,12,13,14,15,16,17,18]. According to the NOx storage mechanism proposed by Fridell and co-workers [5], NO_2_, coming from the oxidation of NO onto Pt active sites, is mainly adsorbed on BaO forming surface nitrates by a disproportionation reaction that involves the release of one molecule of NO for every three molecules of NO_2_ adsorbed (1).

(1) $$3{\mathrm{NO}}_{2}+\mathrm{BaO}\to \mathrm{Ba}{\left({\mathrm{NO}}_{3}\right)}_{2}+\mathrm{NO}$$

Recently, Broqvist et al. [6] suggested, supported by DFT calculations, that NO_2_ adsorption on a BaO surface can take place either on oxygen surface sites or on Ba (II) sites forming nitrates and nitrites, respectively. The generation of the nitrate–nitrite pairs involves a partial oxidation of the surface, and finally, only nitrates would be the unique ending species present on the surface. However, NOx in exhaust gases is mainly present as a NO/O_2_ mixture, which suggests that this route, which only considers NO_2_ adsorption, might not be the main one for the NOx storage mechanism. Recently, based on transient response experiments and operando FTIR studies, Lietti and co-workers [11] demonstrated the existence of the called “nitrite route” for NOx storage in NO/O_2_ atmosphere. These authors concluded that, firstly, at low temperature, nitrites can be formed on the BaO surface after the NO oxidation at Pt-BaO boundaries. At high temperature, this route will lead to subsequent nitrites to nitrates formation.

Using perovskite mixed oxides, Hodjati et al. [19] and Milt et al. [20] proposed that NOx was adsorbed on BaSnO_3_ and BaCoO_3_ perovskites forming barium nitrates, respectively. These nitrates could decompose at high temperature in an oxidizing atmosphere, allowing the regeneration of the perovskite; however, the high temperature for nitrate desorption induced the segregation of a low fraction of B cation oxide from the perovskite lattice. Abrahamsson et al. [21] concluded (from DFT results) that NO_2_ was more strongly adsorbed than NO on ATiO_3_ perovskite surfaces, but NO and NO_2_ co-adsorption enhanced the stability of the ad-species due to nitrite-nitrate pair formation. Additionally, López-Suárez et al. [22], using SrTi_1-x_Cu_x_O_3_ perovskites, observed that NOx chemisorption could take place by adsorption of NO as nitrites and/or oxidation of NO to NO_2_ and subsequent chemisorption as nitrites/nitrates on the perovskite surface.

In a previous study [23], the partial substitution of Ti by Cu in a BaTiO_3_ perovskite allows obtaining a free-noble-metal catalyst (BaTi_0.8_Cu_0.2_O_3_) with a NOx Storage Capacity (NSC) at 420 °C similar to that shown by platinum-based catalysts (around 300 µmol/g.cat), which could be proposed as a potential component of high-temperature LNT systems for lean burn engines, such as Gasoline Direct Injection engines. The present paper aims to elucidate a mechanism for NOx storage on this free-noble-metal catalyst (BaTi_0.8_Cu_0.2_O_3_). Thus, the aim of this study is to determine the species formed under NO and NO/O_2_ atmospheres (under temperature programmed and isothermal conditions) by in situ DRIFTS, as well as to correlate the results with the NOx storage capacity of the catalyst. Finally, other ex situ characterization techniques have been used to try to identify the active phases for NOx storage in the BaTi_0.8_Cu_0.2_O_3_ catalyst.

## 2. Materials and Methods

### 2.1. Synthesis and Characterization of Catalysts

BaTi_0.8_Cu_0.2_O_3_ catalyst was prepared using the Pechini sol-gel method [24] modified to be used in an aqueous media [24,25,26] as detailed elsewhere [23]. In brief, the titanium isopropoxide (Ti) was hydrolyzed and the resulting specie was solved in an aqueous solution of citric acid (CA) (Ti:CA = 1:2) and hydrogen peroxide (Ti:H_2_O_2_ = 2:1), forming a citrate-peroxo-titanate (IV) complex. Afterwards, the pH was dropwise adjusted to 8.5 with NH_3_, and the barium (Ba:Ti = 1:1) and copper precursors, corresponding to the stoichiometry (BaTi_0.8_Cu_0.2_O_3_), were added. The solution was kept at 65 °C for 5 h, until a gel was obtained. Then, the sample was dried at 90 °C for 24 h and, finally, calcined at 850 °C for 6 h.

The crystalline structure of the fresh and used catalyst was obtained by X-ray diffraction (XRD). X-ray diffractograms were recorded in a Brucker D8-Advance diffractometer (Berlin, Germany), using CuKα (0.15418 nm) radiation. Diffractograms were registered from 20 to 80° 2θ angles, with a step of 0.02° and a time per step of 3 s.

XPS were recorded using a K-Alpha Photoelectron Spectrometer from Thermo-Scientific with an AlKα (1486.6 eV) radiation source. The pressure of the analysis chamber was maintained at 5 × 10^−10^ mbar during XPS spectra recording. The binding energy (BE) and kinetic energy (KE) scales were adjusted setting the C1s transition at 284.6 eV, and the BE and KE values were then determined with the peak-fit software of the spectrometer.

Infrared spectroscopy analysis of the fresh and used samples was performed using a JASCO FT/IR 4700 spectrometer fitted with a DLaTGS detector and an ATR Specac Golden Gate accessory. From these spectra, a semi-quantitative estimation of the percentage of barium carbonate in the fresh and used catalyst was carried out. For this analysis, BaTi_0.8_Cu_0.2_O_3_ sample powder was mixed in an agate mortar with BaSO_4_ using a 1:1 ratio. The spectra of the resulting powder mixture were recorded in the 4000–400 cm^−1^ range, with a 1 cm^−1^ resolution and as an average of 100 scans. From these spectra, the relationship between the intensities of the broad barium sulfate band, at ∼1060 cm^−1^, and the small peak due to BaCO_3_, at 860 cm^−1^, (I_BaSO4,1060cm−1_/I_BaCO3860cm−1_) was calculated. The percentage of BaCO_3_ in the BaTi_0.8_Cu_0.2_O_3_ catalyst was estimated, using a calibration curve drawn following identical procedure with I_BaSO4,1060cm−1_/I_BaCO3860cm−1_ values measured from BaCO_3_:BaSO_4_ mixtures with different BaCO_3_:BaSO_4_ ratios.

The percentage of BaCO_3_ present in fresh and used catalyst was also determined by thermogravimetric analysis using 30 mg of the fresh and used catalysts which was heated at 10 °C/min from 25 to 900 °C under a He flow (100 mL/min, P_total_ = 1 atm). These experiments were carried out in a TG-DTA device from Mettler-Toledo (model TGA/SDTA851e/LF/1600), fitted with a QMS (Quadrupole Mass Spectrometer, Pfeiffer Vacuum model Thermostar GSD301T).

### 2.2. NOx Storage Tests

NOx storage tests were performed in a fixed-bed reactor at atmospheric pressure under a gas flow of 500 mL/min (GSHV = 30,000 h^−1^) containing either 500 ppm NO + 5% O_2_ or 500 ppm NO in N_2_. The catalytic bed was composed of 80 mg of catalyst and 320 mg of SiC. The gas composition was monitored by NDIR-UV gas analyzers for NO, NO_2_, CO, CO_2_ and O_2_ (Rosemount Analytical Model BINOS 1001, 1004 and 1000). Temperature programmed reactions (10 °C/min from room temperature to 800 °C) were performed using the as-prepared catalyst. NOx conversion profiles were determined as a function of temperature using the following Equation (2):

(2) $$\mathrm{NOx}\;\mathrm{conversion}\;\left(\%\right)=\frac{\mathrm{NOx}{,}_{\mathrm{in}}-\mathrm{NOx}{,}_{\mathrm{out}}}{\mathrm{NOx}{,}_{\mathrm{in}}}\times 100$$

where “NOx_,in_” is the concentration of NOx (= NO + NO_2_) fed to the reactor, while “NOx_,out_” is the concentration of NOx measured by the analyzers at the exit of the reactor.

The NSC was determined by carrying out ten consecutive storage–regeneration cycles at the selected temperatures by using the following procedure: (i) during the lean cycle (5 min) a gas flow (500 mL/min (GSHV = 30,000 h^−1^)) composed of 500 ppm NO and 5% O_2_ balanced with N_2_ was fed through the reactor while a rich gas flow (10% H_2_ as model reductant in N_2_ balance) was fed through a bypass path; (ii) at the required time, the gas paths were switched and during 3 min, the rich gas flow was fed into the reactor to regenerate the catalyst (rich cycle). This procedure was also carried out using a reactor without catalyst (400 mg of SiC), to determine the NOx,inlet response of the gas analyzers. Please note that, although the time for rich and lean cycles is far from that of the real conditions, it allows determining the ability of the catalysts to store NOx.

The NSC was calculated as the difference between the NOx signal when the reactor is filled with SiC and the NOx signal when the reactor is filled with the catalyst, by using Equation (3):

(3) $$\mathrm{NSC}\;=\int_{t_{0}}^{t_{f}}{\mathrm{NO}}_{X,\;\mathrm{inlet}}\left(t\right)-{\mathrm{NO}}_{X,\exp}\left(t\right)\mathrm{dt}$$

where “NOx_,inlet_” is the concentration of NOx (= NO + NO_2_) measured for the SiC filled reactor, and “NOx_,exp_”, is the concentration of NOx during the NOx storage test.

### 2.3. In Situ Diffuse Reflectance Infrared Fourier Transform Spectroscopy (DRIFTS) Experiments

The analysis of species adsorbed on the catalyst surface during the NOx storage experiments (both under temperature programmed reaction and isothermal conditions) was carried out using a Shimadzu IRTracer-100 FTIR Spectrophotometer fitted with MCT detector. The instrumentation included Praying Mantis optics and a Harrick DRIFTS cell, which allowed heating of the samples to 550 °C. Spectra were recorded with a 4 cm^−1^ resolution from 4000 to 400 cm^−1^ and as an average of 16 scans. All catalysts were pretreated in the DRIFTS cell, at 450 °C for 30 min in a 500 ppm NO + 5% O_2_ + N_2_ atmosphere (100 mL/min), followed by 30 min in 10% H_2_ + N_2_ at the same temperature (regeneration step). Afterwards, the samples were cooled down to the desired temperature in N_2_ atmosphere.

For temperature-programmed-reaction experiments, the preconditioned catalyst was heated at 10 °C/min from 50 to 500 °C and an IR spectrum was recorded every 50 °C. Five consecutive NOx storage–regeneration cycles were carried out at 300, 350, 400 and 450 °C with the preconditioned catalyst and using lean (500 ppm NO + 5% O_2_ balanced with N_2_) and rich (10% H_2_ balanced with N_2_) 100 mL/min gas flows, respectively. As this paper is focused on the NOx storage process, only the species formed during the lean cycle was followed by recording an IR spectrum every 30 s while the lean atmosphere was fed into the DRIFTS cell.

## 3. Results

### 3.1. Catalyst Characterization

A detailed discussion of the characterization results of the BaTi_0.8_Cu_0.2_O_3_ has been presented elsewhere [23]. The more relevant characterization results are included in Figure S1a–d and Table S1 in the Supplementary Materials. Briefly, BaTi_0.8_Cu_0.2_O_3_ catalyst synthetized by sol–gel method is a mixed oxide with perovskite structure (XRD) and negligible porosity. The XRD, XPS, and TPR-H_2_ results revealed that copper has been incorporated into the BaTiO_3_ perovskite structure with different electronic interaction with the lattice (and hence with different reducibility). The incorporation of copper into the structure generates oxygen vacancies, feasible active oxygen species for adsorption on the catalyst surface and, it induces both a pseudo distortion of the tetragonal structure (XRD and Raman Spectroscopy) and the segregation of some phases (mainly BaCO_3_ and Ba_2_TiO_4_, but also a minor phase of CuO). As a consequence of all these modifications, active sites for the NO to NO_2_ oxidation and for the NOx storage are created on the copper-doped perovskites [23].

### 3.2. NOx Storage under Temperature-Programmed Reaction Conditions

**Figure 1:**
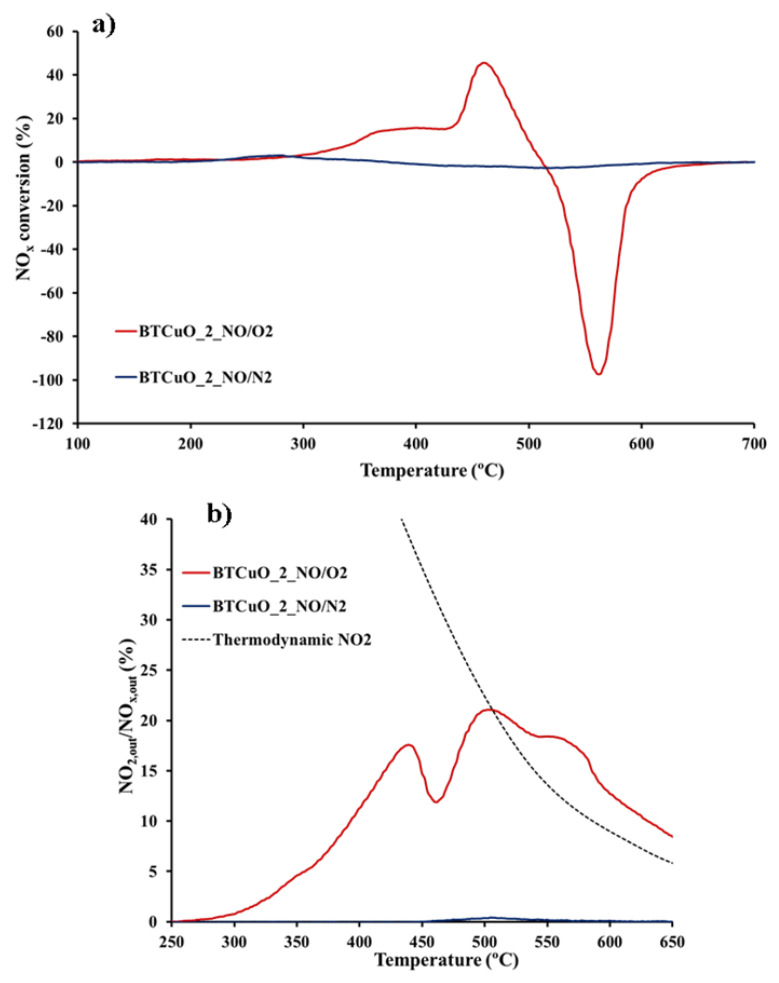
(**a**) NOx conversion and (**b**) NO_2_ generation profiles for BaTi_0.8_Cu_0.2_O_3_ catalyst in 500 ppm NO + 5% O_2_ and 500 ppm NO atmospheres under Temperature Programmed conditions.

Figure 1a,b shows the NOx conversion (calculated from Equation (2)) and the NO_2_ generation profiles obtained for the BaTi_0.8_Cu_0.2_O_3_ catalyst under both NO and NO/O_2_ atmospheres. In these profiles, a positive value of NOx conversion indicates that NOx is stored on the catalyst as the NOx concentration measured by the analyzers is lower than that fed into the reaction system, so, %NOx conversion means %NOx storage. On the contrary, a negative value of NOx conversion reveals that NOx has been desorbed, so in this case, %NOx conversion represents %NOx desorption [23]. A clear influence of the gas phase composition on the NOx conversion profiles is observed. In a NO/O_2_ atmosphere (red line), the NOx conversion profile shows two adsorption maxima around 300–350 °C and 450 °C, and only one NOx desorption peak at 550 °C. However, in NO/N_2_ atmosphere (blue line), an almost flat NOx conversion profile is found, suggesting that adsorption/desorption processes do not takes place under these conditions.

**Figure 2:**
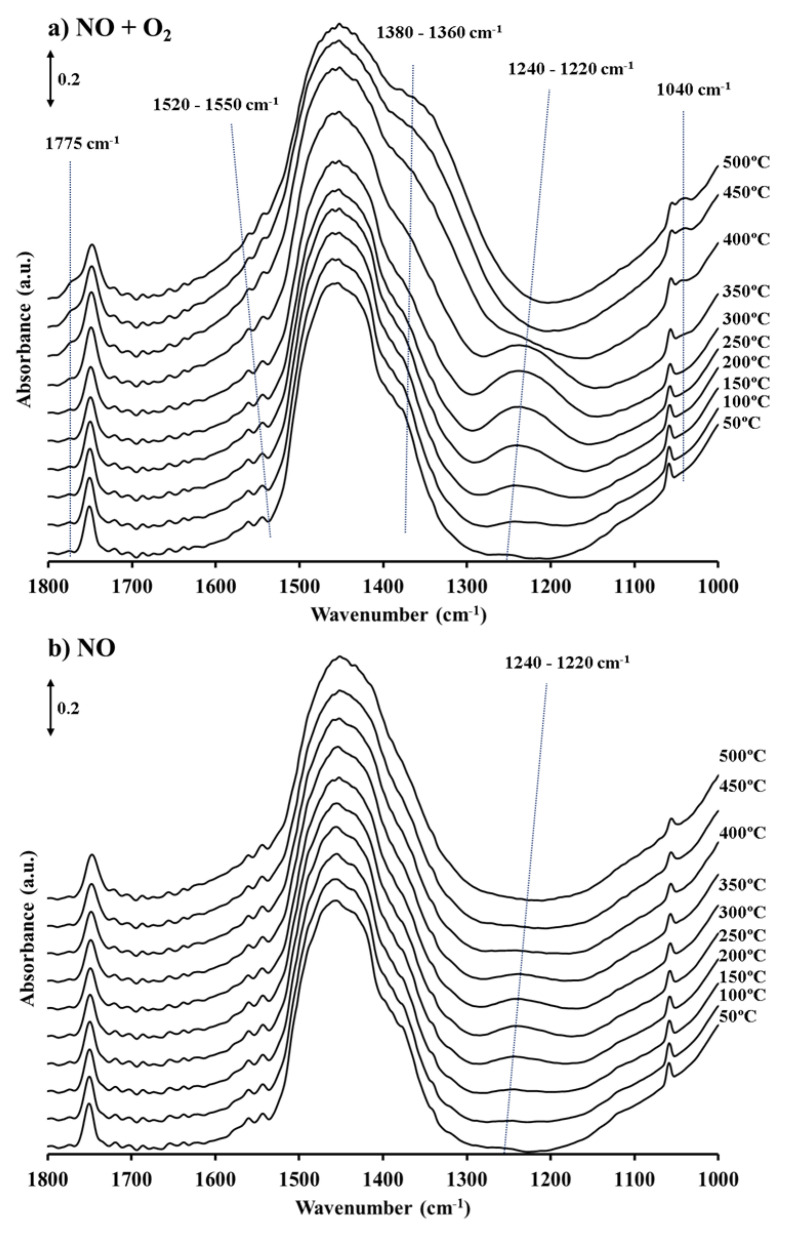
DRIFT spectra recorded under temperature programmed conditions in (**a**) 500 ppm NO + 5% O_2_ and (**b**) 500 ppm NO atmosphere.

To identify the species generated on the BaTi_0.8_Cu_0.2_O_3_ catalyst as a function of temperature and atmosphere composition (NO/N_2_ or NO/O_2_), analogous temperature programmed reaction experiments were performed during in situ DRIFTS evaluation. The DRIFT spectra ranging from 1800 to 1000 cm^−1^ are shown in Figure 2a,b, as it has been widely reported that this region corresponds with the wavenumber range where bands ascribed to nitrite/nitrate species can be identified [27], for instance, ionic (~1380 cm^−1^), monodentate (1530–1480 cm^−1^), bidentate (1565–1500 cm^−1^) or bridged nitrates (1650–1600 cm^−1^), ionic (~1260 cm^−1^), monodentate (1470–1450 cm^−1^) or bridged nitrites (1220–1205 cm^−1^), and nitro compounds (1470–1450 cm^−1^).

All the DRIFT spectra show three main bands corresponding to carbonate groups present on the catalyst [28] (not labeled in Figure 2a,b) at 1055, 1460 and 1760 cm^−1^. The intensity of these bands was not affected by the pre-treatment of the catalyst, suggesting a high stability of these species. Considering that the broad band at 1460 cm^−1^ [29], overlaps with some of the bands corresponding to NOx species and, also, the discrepancies published in literature related to the exact assignment of the different types of nitrate/nitrite species, in the subsequent discussion, the peaks detected in the DRIFT spectra will be assigned as barium nitrites or nitrates, thus, avoiding specific assignations among the different nitrogen species.

Under a NO/O_2_ atmosphere (Figure 2a), a band at 1240–1220 cm^−1^ corresponding to barium nitrites [9,11,17] is identified at 100 °C. The intensity of this band increases with temperature up to 350 °C, when it achieves its maximum intensity. Beyond this temperature, the nitrites band starts to vanish, and new bands grow at 1040, 1360, 1540 and 1775 cm^−1^ ascribed to the formation of barium nitrates [15,30,31]. The latter bands become the only ones identified in the spectra recorded at 450 and 500 °C, confirming that barium nitrate is the final product in the NOx storage process at high temperature. These results totally agree with previous studies concluding that, under a NO/O_2_ atmosphere, the formation of nitrites on catalyst is the main route at low temperature, while at high temperature, these groups are oxidized to nitrates [5,11].

Two main differences are detected when compared the spectra recorded in a NO atmosphere (Figure 2b) with the former one. Firstly, bands ascribed to nitrites (1240–1220 cm^−1^) are identified in spectra of the sample between 100 and 350 °C, but they show significantly lower intensity than under a NO/O_2_ atmosphere (Figure 2a) indicating that in the absence of O_2_, the NOx storage capacity of the BaTi_0.8_Cu_0.2_O_3_ perovskite is much lower or almost negligible, which agrees with NOx conversion profiles (Figure 1b). Secondly, in the DRIFT spectra recorded above 350 °C, nitrite bands disappear (as observed in the presence of O_2_) but peaks corresponding to barium nitrates are not clearly detected. The decrease in the intensity of the nitrite band suggests that these groups are unstable above 350 °C in absence of O_2_ in the atmosphere; however, partial oxidation of nitrites to nitrates may not be ruled out although the nitrate bands could not clearly be detected [29].

### 3.3. NOx Storage under Isothermal Reaction Conditions

Cyclic NOx storage–regeneration experiments were carried out under NO/O_2_ between 300 and 450 °C (temperature range at which the BaTi_0.8_Cu_0.2_O_3_ catalyst shows NOx storage capacity according to Figure 1a).

Table 1 shows the amount of NOx stored (as NSC, NOx Storage Capacity, in µmol/g. catalyst) on the catalyst at different temperatures, calculated as the difference between the NOx signals when the reactor is filled with SiC (inert material) and filled with catalyst.

Please note that, as expected [23], NSC increases with temperature. It is widely accepted that NO_2_ is the main species adsorbed on an NSR catalyst, so, direct correlation exists between NO_2_ generation activity and NSC. In agreement with this, in the BaTi_0.8_Cu_0.2_O_3_ catalyst, the highest NSC is achieved at the temperature range in which this catalyst shows its highest NO to NO_2_ oxidation activity (Figure 1b). It is remarkable that the NSC featured by the BaTi_0.8_Cu_0.2_O_3_ catalyst is within the range of values expected for NSR application, even though the temperature is higher than those reported for model NSR catalysts, for this reason, it has been suggested that this new material might be of interest for high-temperature applications, as in GDI engines [23].

Analogous cyclic NOx storage–regeneration experiments were carried out with the BaTi_0.8_Cu_0.2_O_3_ catalyst in a DRIFTS cell to determine the species formed during NOx storage. The DRIFT spectra were registered at 30 s time intervals, once the lean atmosphere (composed of 500 ppm of NO, 5% of O_2_ in N_2_) was fed. To ensure catalyst regeneration, a 10% H_2_ in N_2_ (rich conditions) was used during the regeneration cycle.

**Figure 3:**
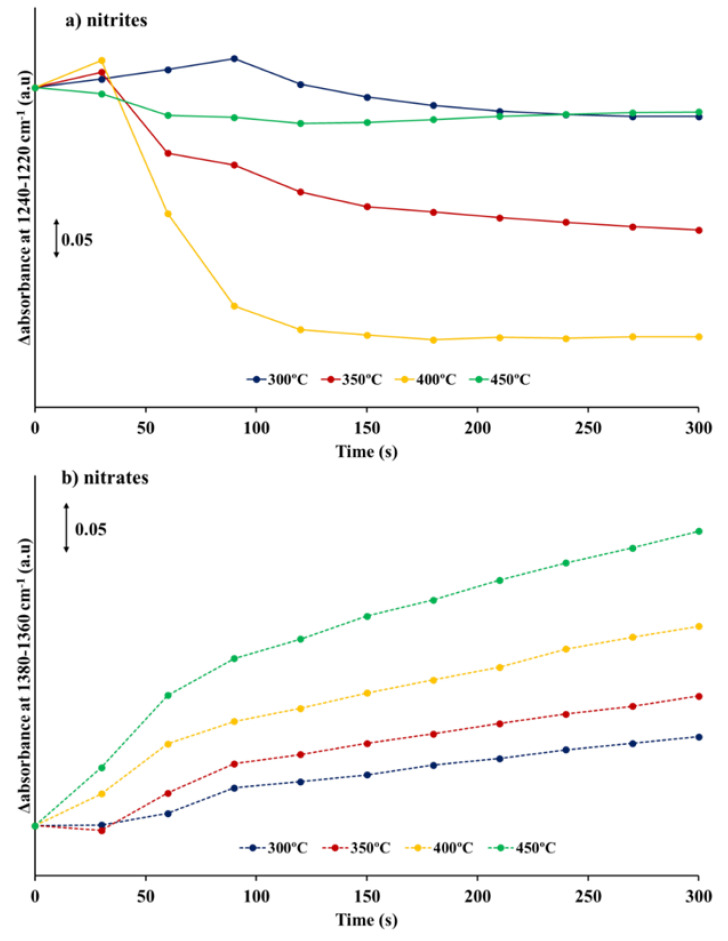
Evolution of intensity of absorbance bands ascribed to (**a**) nitrites (1240–1220 cm^−1^) and (**b**) nitrates (1380–1360 cm^−1^) obtained from the DRIFT spectra shown in Figure S2a–d.

Figure S2a–d (Supplementary Materials) shows the DRIFT spectra obtained during the NOx storage cycles. These spectra show that, at any temperature, the amount of NOx species on the catalyst surface increases with time on NO/O_2_ stream. However, due to the short time of the storage-cycle and likely overlapping with the carbonate bands, some of the signals ascribed to nitrates and nitrites might be hard to identify. Thus, in order to clarify the analysis, only the evolution in the intensity of two selected bands was followed during NOx exposure: (i) 1240–1220 cm^−1^ for nitrites, and (ii) 1380–1360 cm^−1^ for nitrates. In Figure 3a,b, the difference between the absorbance values (after baseline correction) recorded every 30 s and the absorbance value registered once the switch from rich to lean conditions is carried out (i.e., at t = 0 s) is shown as a function of time and for each reaction temperature (300, 350, 400 and 450 °C). As observed under temperature-programmed reaction conditions, at low temperature (300 °C) the NOx storage takes place forming nitrites and nitrates. Thus, at 300 °C, the intensity of the nitrites band (Figure 3a) increases during the early stages of exposure to NO/O_2_ (~90 s), but longer time on stream involves a depletion of nitrites followed by an increase of the nitrate bands (Figure 3b). As the storage temperature increases, two clear effects can be noticed:

1. At 350 and 400 °C, the lifetime of the nitrite species, formed at the beginning of the NOx storage cycle, becomes shorter. Thus, the intensity of the nitrite band increases until 30 s of NO/O_2_ exposition and, afterwards, the nitrite band intensity drastically drops. This trend suggests that the oxidation of nitrites to nitrates becomes faster at these temperatures than at 300 °C. In fact, at 450 °C, nitrites are not detected even at the very beginning of the NOx storage step.
2. A significant increase in the intensity of the nitrates band (Figure 3b) is detected as the storage temperature rises, which can be directly related to the increase of the NSC (Table 1).

Thus, in situ DRIFTs results obtained under isothermal conditions confirms that, between 300 and 400 °C, nitrites and nitrates coexist on the catalyst but, as the temperature increases, nitrite oxidation to nitrates become faster and consequently, only nitrates are detected at higher temperature (450 °C).

### 3.4. Identification of Active Phase for NOx Storage

**Figure 4:**
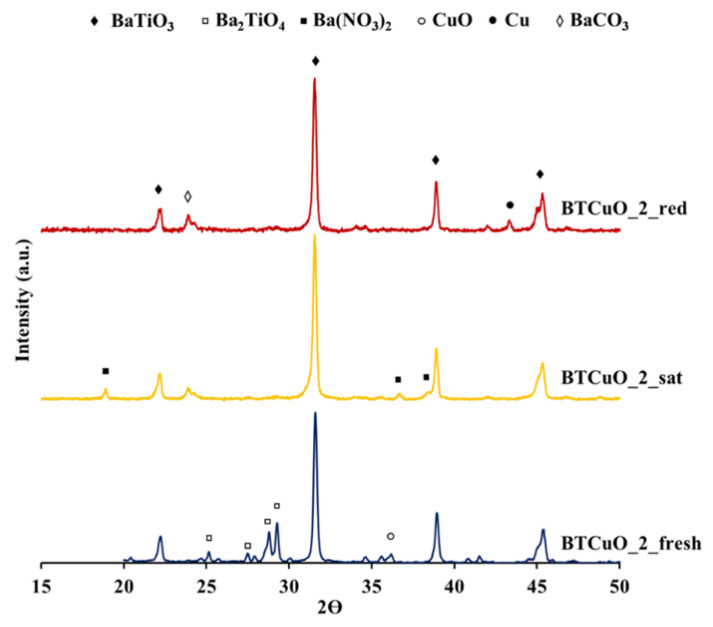
XRD patterns recorded for the as-prepared BaTi_0.8_Cu_0.2_O_3_ catalyst (BTCuO_2_fresh), after 1 h of NOx exposure in 500 ppm NO + 5% O_2_ in N_2_ atmosphere (BTCuO_2_sat) and finally after regeneration in 10% H_2_/N_2_ atmosphere (BTCuO_2_red).

XRD was used to determine any effect of NOx storage on the perovskite structure of the BaTi_0.8_Cu_0.2_O_3_ catalyst. Additionally, this technique may help to identify the role of the BaCO_3_ or Ba_2_TiO_4_ segregated phases (identified in the XRD patterns of the fresh catalyst) in the NOx storage process. Figure 4 compares the XRD pattern of the fresh BaTi_0.8_Cu_0.2_O_3_ catalyst with the diffractograms recorded after two different ex-situ pretreatments: (i) 1 h exposure to NOx at 400 °C using an atmosphere composed of 500 ppm NO + 5% O_2_ balanced with N_2_, to simulate catalyst saturation (called BTCuO_2_sat), and (ii) 1 h under an NO/O_2_ atmosphere at 400 °C followed by 1 h under rich conditions (10% H_2_/N_2_), for reproducing the regenerated catalyst, called BTCuO_2_red. It is worth mentioning that, for this analysis, longer time pretreatments have been used since XRD characterization does not allow the detection of surface phases, therefore, under these new conditions, the generation of bulk and crystalline phases was guaranteed.

The XRD pattern of the fresh BaTi_0.8_Cu_0.2_O_3_ catalyst shows that tetragonal BaTiO_3_ perovskite is the main phase in this catalyst, while the incorporation of copper induces the segregation of mainly BaCO_3_ and Ba_2_TiO_4_, but also CuO phases [23] (denoted as BTCuO_2 in Figure S1a in Supplementary Materials). After the first 1 h NOx storage pretreatment (BTCuO_2_sat), the perovskite structure remains as the main phase; however, the peaks corresponding to the Ba_2_TiO_4_ phase disappear and new diffraction signals ascribed to Ba(NO_3_)_2_ [32] and BaCO_3_ are identified. These changes in the diffraction pattern suggest that the segregated Ba_2_TiO_4_ phase is acting as an NOx storage phase in NO/O_2_ atmosphere, forming barium nitrates. It is worth mentioning that, although BaCO_3_ is observed in the XRD pattern of the as prepared catalyst, the higher intensity of the carbonate reflection for BTCuO_2_sat may be attributed to further carbonation of barium nitrates during the exposure of the catalyst to atmospheric CO_2_ before recording the XRD pattern.

In the XRD pattern registered after catalyst regeneration (BTCuO_2_red), the perovskite structure is still the main phase, so, even after a regeneration cycle, the structure does not collapse. In this pattern, peaks ascribed to barium nitrate are not identified, confirming that nitrates are removed during the regeneration cycle. However, as peaks corresponding to the Ba_2_TiO_4_ phase are neither detected, it seems that the regeneration of the Ba_2_TiO_4_ phase does not occurs after barium nitrate removal. Additionally, the increase in the intensity of the BaCO_3_ peaks with respect to the BTCuO_2_sat suggests that barium nitrate decomposition leads to the formation of BaO phase, which is identified as barium carbonate by XRD due to the carbonation after exposure to the atmospheric CO_2_. Please note that a new diffraction peak assigned to metallic copper is also observed in the BTCuO_2_red pattern. Considering the rich cycle conditions of this pretreatment (10% H_2_/N_2_ at 400 °C), this peak is consistent with catalyst reducibility [23] (Figure S1d in Supplementary Information).

The percentage of barium carbonate for the BaTi_0.8_Cu_0.2_O_3_ catalyst at the different stages of the NOx storage–regeneration process was assessed using Attenuated Total Reflectance spectroscopy (ATR), thermogravimetric analysis (TGA) and XPS techniques. For this evaluation, in order to conditioning the catalyst, three different pretreatments were performed: (i) five NOx storage–regeneration cycles at 400 °C called BTCuO_2_NSR, (ii) five NOx storage–regeneration cycles at 400 °C followed by 1 h in NO/O_2_ atmosphere at the same temperature for simulating catalyst saturation, called BTCuO_2_sat, and (iii) five NOx storage–regeneration cycles at 400 °C followed by 1 h in NO/O_2_ atmosphere and, subsequently, 1 h regeneration under rich conditions (to reproducing a regenerated catalyst surface situation), called BTCuO_2_red. The results of this estimation of barium carbonate by these three techniques are listed in Table 2.

**Figure 5:**
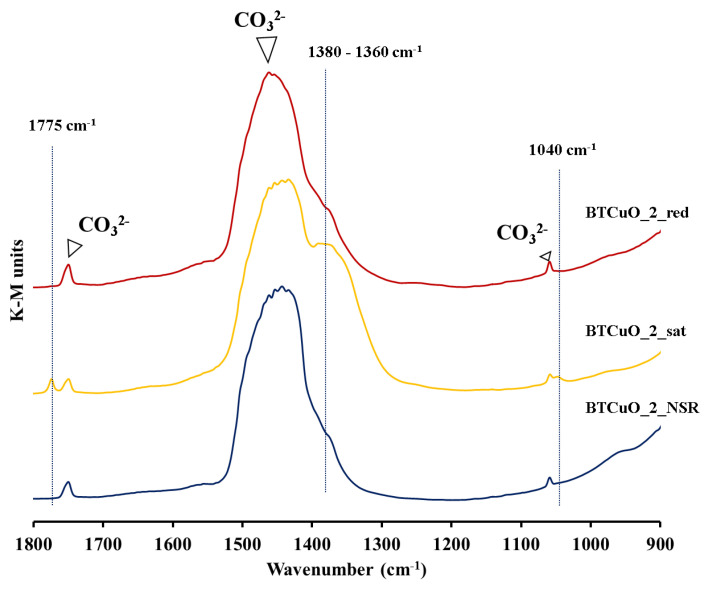
DRIFT spectra for the BaTi_0.8_Cu_0.2_O_3_ catalyst after 5 NOx storage–reduction cycles (BTCuO_2_NSR), followed by 1 h NOx exposure experiment in 500 ppm NO + 5% O_2_ in N_2_ (BTCuO_2_sat) and finally regeneration in 10% H_2_/N_2_ (BTCuO_2_red).

In the DRIFT spectra of the fresh BaTi_0.8_Cu_0.2_O_3_ catalyst, three bands at 1055, 1460 and 1760 cm^−1^, assigned to barium carbonate, are identified. Figure 5 shows the DRIFT spectra recorded after the three described pretreatments. In agreement with XRD, carbonates are still detected for BTCuO_2_sat and for BTCuO_2_red proving the high stability of these species under experimental conditions. However, bands corresponding to nitrates (blue dotted lines) are only detected for BTCuO_2_sat, confirming that NOx stored on the catalyst is taking place by forming nitrates as final product. As these nitrate bands are not identified after the NOx storage–regeneration cycles (i.e., reduction pretreatments), the total regeneration of the catalyst is confirmed.

Despite significant visual changes in the intensity of the carbonates band at ~1460 cm^−1^ are not observed in the spectra (Figure 5), ATR spectroscopy has been used for estimating the percentage of carbonate remaining after every pretreatment (see Experimental Section for details). Data in Table 2 show that the percentage of barium carbonate decreases after NOx uptake experiments, confirming that carbonates are removed from the catalyst during Nox exposure because they are displaced by NOx, leading to nitrates as a final product (Figure 5). After catalyst regeneration (BTCuO_2_red), a recovery of the carbonate percentage due to the adsorption of atmospheric CO_2_ on the BaO phase, formed from barium nitrate decomposition, is found.

**Figure 6:**
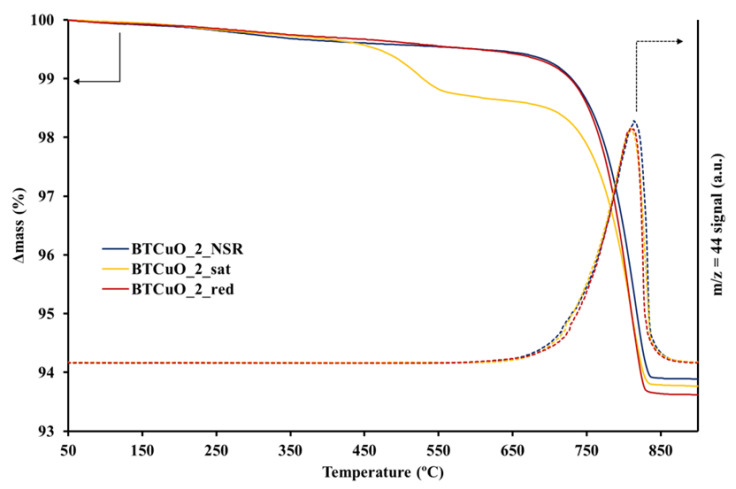
TGA and *m*/*z* = 44 profiles in He of the BaTi_0.8_Cu_0.2_O_3_ catalyst after 5 NOx storage-reduction cycles (BTCuO_2_NSR), followed by 1 h NOx exposure to 500 ppm NO + 5% O_2_ in N_2_ (BTCuO_2_sat) and finally reduced in 10% H_2_/N_2_ (BTCuO_2_red).

The weight loss profiles, obtained in a TG experiment carried out up to 900 °C and using 100 mL/min He gas flow, are shown in Figure 6. The weight losses at high temperature (maximum slope at approximately 815 °C) are listed in Table 2. This weight loss is due to the BaCO_3_ decomposition to BaO and CO_2_, since an intense signal corresponding to CO_2_ release (*m*/*z* = 44 and 28) was detected by a QMS at this temperature (not shown). Please note that in the TGA profile of the BTCuO_2_sat catalyst, a weight loss is observed between 450 and 550 °C, which corresponds to desorption of the previously adsorbed NOx (Figure 1) during the NOx storage pretreatment (a release of NO (*m*/*z* = 30) and O_2_ (*m*/*z* = 32), (not shown), was detected by QMS at that temperature). In Table 2, the trend in the BaCO_3_ percentage determined by TGA after the different pretreatments is similar to that shown by ATR spectroscopy, i.e., a decrease in barium carbonate percentage after exposure to NOx, and an increase in this value after the regeneration pretreatment. This fact supports carbonates being released from the BaTi_0.8_Cu_0.2_O_3_ catalyst during NOx storage and regenerated after CO_2_ atmospheric exposure, once NOx is removed from the catalyst after the regeneration pretreatment.

The atomic percentage of Ba, Ti, O, Cu and C on the surface of the BaTi_0.8_Cu_0.2_O_3_ catalyst after the three pretreatments was determined by XPS from the signals corresponding to the Ba3d_3/2_, Ti2p_3/2_, O1s, and Cu2p_3/2_, and C1s transitions, respectively. From the Ba and O profiles, the presence of carbonates on the catalyst surface is proven. In general, the C1s transition is used in XPS analysis as a standard reference for setting the XPS transitions of the other elements, since carbon (hydrocarbon) is a common impurity in most of the materials under ultra-high-vacuum conditions (284.6 eV) [33]. For the BaTi_0.8_Cu_0.2_O_3_ catalyst, the C1s transition showed a shoulder at ~289–290 eV (Figure S3 in Supplementary Information) which is associated with the presence of carbonate groups [34]. Thus, the percentage of C due to carbonates was calculated from the area of this peak, after recalculating the quantities of all the elements present in the sample by correcting the contribution of carbon impurities (Table 2). As observed by ATR and TGA (which determines bulk carbonates), the percentage of C ascribed to the presence of surface is lower for BTCuO_2_sat than for BTCuO_2_NSR and grows during regeneration (BTCuO_2_red).

## 4. Discussion

The aim of this study is to analyze the NOx storage process on a BaTi_0.8_Cu_0.2_O_3_ catalyst from a mechanistic point of view. The results show that, as a model NSR catalyst [7], nitrites and nitrates are formed in the BaTi_0.8_Cu_0.2_O_3_ catalyst when exposed to a NO/O_2_ atmosphere.

The characterization results revealed that the introduction of copper into the perovskite lattice originates a pseudo distortion of the structure, but also the segregation of phases such as Ba_2_TiO_4_ and BaCO_3_. The formation of Ba_2_TiO_4_ is identified as an intermediate in the synthesis of BaTiO_3_ from BaCO_3_ and TiO_2_ reaction by ceramic method [35,36,37,38,39]. Some of these studies conclude that Ba_2_TiO_4_ is formed in the reaction zone between TiO_2_ and BaCO_3_ such that at the end of the process a thin layer on the BaTiO_3_ surface may remain. In agreement with this conclusion, the XPS (Cu + Ti)/Ba ratio in Table S1 (calculated from the atomic percentage of Cu, Ti and Ba) shows the surface barium enrichment due to the presence of Ba_2_TiO_4_ as a segregated phase [23].

XRD patterns (Figure 4) of sample after exposure to a NO/O_2_ atmosphere at 400 °C, reveal that Ba_2_TiO_4_ mixed oxide acts as an active phase for NOx storage because, at this temperature and after 1 h exposure, peaks corresponding to this phase totally disappear and new peaks assigned to Ba(NO_3_)_2_ are detected [32]. Szanyi et al. [40] analyzed by TR-XRD the NO_2_ uptake process on a BaO/Al_2_O_3_ catalyst after 5 min of exposure to NO_2_ at different temperatures. In this analysis, they detected peaks corresponding to barium nitrates and the decrease in the intensity of BaO diffraction peaks during the experiment. On the basis of this result, the authors concluded that NO_2_ storage leads to the formation of bulk barium nitrates that transforms the BaO phase. Please note that, at 400 °C, the BaTi_0.8_Cu_0.2_O_3_ catalyst shows a high NO to NO_2_ oxidation activity (Figure 1b); therefore, the transformation of Ba_2_TiO_4_ into barium nitrates seems to take place when high NO_2_ concentrations are present in the gas phase. This suggests that NO_2_ is being consumed by following Reaction (4):

(4) $${\mathrm{Ba}}_{2}{\mathrm{TiO}}_{4}+2{\mathrm{NO}}_{2}+\frac{1}{2}O_{2}\to \mathrm{Ba}{\left({\mathrm{NO}}_{3}\right)}_{2}+{\mathrm{BaTiO}}_{3}$$

Templeton and Pask [37] and Beauger et al. [41] demonstrated that the CO_2_ partial pressure has a key role on the presence of the intermediate Ba_2_TiO_4_ phase during BaTiO_3_ synthesis by the ceramic method. Both studies concluded that CO_2_ reacts with the intermediate Ba_2_TiO_4_ at high temperatures leading to the formation of barium carbonate and barium titanate as final products. Considering the competition between CO_2_ and NO_2_ for the basic oxide sites of a NSR model catalyst [42,43], despite different acidities of CO_2_ and NO_2_, it may be assumed that the NOx storage follows the previously proposed Reaction (4).

After the regeneration pretreatment, XRD peaks of the barium nitrate disappears due to desorption of the previously stored NOx species, thus, indicating that the removal of them from the catalyst is achieved during the rich cycle. However, as the diffraction peaks corresponding to the Ba_2_TiO_4_ phase are not detected after nitrate decomposition, it seems that Ba_2_TiO_4_ is not formed again. Accordingly, peaks ascribed to the presence of BaCO_3_ phase are clearly identified with a higher intensity than that observed after the NOx adsorption process. By TR-XRD [40], it has been demonstrated that barium nitrate decomposition in an inert atmosphere leads to the formation of barium oxide. As BaO is readily carbonated due to the exposure to air, it can be concluded that, after the regeneration cycle, barium oxide is formed as a primary product of barium nitrate decomposition. During the NOx storage experiment under temperature programmed conditions, NO and NO_2_ are detected at NOx desorption temperatures (Figure S4 in Supplementary Information). Thus, the following two reactions account for nitrate decomposition pathways (5a,b)

(5a) $$\mathrm{Ba}{\left({\mathrm{NO}}_{3}\right)}_{2}\to \mathrm{BaO}+2\mathrm{NO}+\frac{3}{2}O_{2}$$

(5b) $$\mathrm{Ba}{\left({\mathrm{NO}}_{3}\right)}_{2}\to \mathrm{BaO}+2{\mathrm{NO}}_{2}+\frac{1}{2}O_{2}$$

Reactions (5a,b) show the barium nitrate decomposition process and, consequently, the barium oxide formation, which is indirectly identified by XRD as barium carbonate. However, it cannot be ruled out that, due to the presence of H_2_ during the reduction pretreatment, NO and NO_2_ can be also partially reduced to nitrogen instead of being only desorbed in the form of nitrogen oxides.

In summary, it can be concluded that Ba_2_TiO_4_ is an active phase for NOx storage from a NO/O_2_ atmosphere, forming barium nitrate and barium titanate. However, this is an irreversible process that leads to BaO formation after nitrate decomposition, instead of Ba_2_TiO_4_ regeneration. Thus, BaO, which is originated from Ba_2_TiO_4_, seems to be the active phase for NOx storage in this catalyst when performing in cycling conditions after first NOx adsorption step. Therefore, during the first NOx storage cycle using the as-prepared BaTi_0.8_Cu_0.2_O_3_ catalyst, Ba_2_TiO_4_ would act as a NOx storage site during the rich stage and, during regeneration, BaO would be formed by barium nitrate decomposition which becomes the active site for NOx storage in subsequent NOx storage–regeneration cycles.

Based on these conclusions, the relevance of the BaO phase generated after the removal of NOx from the catalyst surface as a NOx storage site was analyzed in detail. For this purpose, the changes in the percentage of barium carbonate present in the catalyst after the different pretreatments (after five NOx storage–reduction cycles at 400 °C, after 1 h NOx exposure in NO/O_2_, and also after the reduction of the saturated catalyst under rich conditions for 1 h) was determined by ATR, TGA and XPS. BaCO_3_ was selected from these analyses as it is formed during exposure of pretreated catalyst to atmospheric air [44]. Even though these techniques can be considered as reliable for carbonate identification purposes, Blanco-López et al. [45] demonstrated that it is difficult to assess quantitatively the amount of BaCO_3_ in BaTiO_3_ at low carbonate levels. Accordingly, only the changes in the percentage of carbonate from a semi-quantitative point of view have been discussed. The percentage of barium carbonate measured by ATR, TGA and XPS, and it is worth indicating that these three values are different for the following reasons: (i) only surface carbonate is determined by XPS, (ii) bulk carbonate is measured by TGA, and (iii) the amount of carbonate estimated by ATR could be surface or bulk depending on the experimental conditions (light-beam penetration). Despite of the differences between these three techniques, all data obtained from them (included Table 2) reveal that the percentage of barium carbonate in the BaTi_0.8_Cu_0.2_O_3_ catalyst is lower after NOx saturation (long NOx adsorption cycle) than after NOx storage (short NOx adsorption cycle), which indicates that during adsorption process, NOx displace carbonates generating nitrates that, at the end, are detected by DRIFTS (Figure 5). Please note that these nitrates are not completely removed from the catalyst due to exposure to atmospheric CO_2_, indicating their high stability. After catalyst reduction, in 10% H_2_/N_2_, nitrates are removed, and the catalyst is regenerated. As a consequence, barium oxide is formed which is subsequently carbonated due to the adsorption of CO_2_ from the exposition to atmospheric air [44]. In ATR, TGA and XPS data this fact is translated in an increase in the barium carbonate content after the 10% H_2_/N_2_ regeneration step.

In brief, as a result of the incorporation of copper into the perovskite structure, Ba_2_TiO_4_ is generated which, after NOx storage–regeneration cycles, is converted to BaO that becomes relevant for NOx storage in the BaTi_0.8_Cu_0.2_O_3_ catalyst. Obviously, NOx storage on the perovskite cannot be ruled out (considering that NO_2_ storage on barium perovskites has been reported [20,21,46]), but the low specific surface area of the catalyst under study and also the reported negative effect that the formation of Ba_x_T_iy_O_z_ perovskite has on the NSC of a model BaO/TiO_2_/Al_2_O_3_ [47] support that the role of the perovskite on NOx storage is not significant.

**Figure 7:**
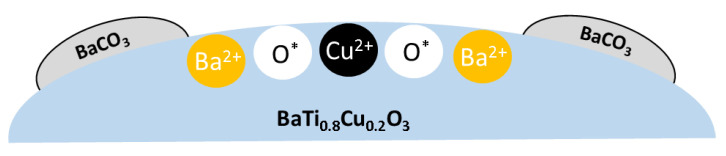
Composition of the BaTi_0.8_Cu_0.2_O_3_ perovskite-type catalyst under reaction conditions.

According to the literature, a large controversy exists related to the formation of Ba_2_TiO_4_/BaCO_3_/BaO as a thin layer on the surface or as discrete particles during BaTiO_3_ synthesis. In analogous TG experiments, Blanco-López et al. [45] did not identify any weight loss associated with the decomposition of surface BaCO_3_ in BaTiO_3_ mixed oxides at the relatively low temperatures observed in this study. They concluded that barium carbonate decomposition is controlled by the partial pressure of CO_2_ at those temperatures [37] and it may be assumed to be a very slow process that could not be properly identified by TGA. Piacentini and co-workers [48] demonstrated that the high amounts of barium oxide lead to the formation of bulk-like barium carbonate, with high thermal stability and low activity for NOx storage. In BaTi_0.8_Cu_0.2_O_3_ catalyst, the low temperature for barium carbonate decomposition and the high NOx storage capacity indicate that this barium carbonate is very reactive, so it should be located at the catalyst surface. On the basis of the above results, a representation of the phases present in the BaTi_0.8_Cu_0.2_O_3_ catalyst is shown in Figure 7.

Once the active phases have been identified, the NOx storage mechanism can be carefully addressed. Two different temperature regions can be distinguished in the in situ DRIFTS experiments carried out under temperature-programmed conditions (Figure 2). Below 350 °C, NOx is stored on the catalyst as nitrites, while above this temperature, nitrites disappear and bands corresponding to nitrates are detected. This trend is in agreement with the two proposed routes for the NOx storage on alkaline oxides [5,10,11]: (i) adsorption of NO and fast oxidation by O_2_ forming nitrites that are subsequently oxidized to nitrates, called the “nitrite route”, and (ii) the formation of nitrates through the adsorption of NO_2_ (obtained from NO oxidation to NO_2_), called the “nitrate route”.

As above 350 °C, BaTi_0.8_Cu_0.2_O_3_ shows its highest NO to NO_2_ oxidation activity (Figure 1b), it can be assumed that the NOx storage mechanism proceeds as follows: (i) oxidation of NO to NO_2_ on the perovskite surface (6), since the copper present in the catalyst is active for NO to NO_2_ oxidation under an NO/O_2_ atmosphere, followed by (ii) NO_2_ adsorption as nitrates on the barium oxide located at the BaTi_0.8_Cu_0.2_O_3_ catalyst surface (7):

(6) $$\mathrm{NO}+\frac{1}{2}O_{2}\to {\mathrm{NO}}_{2}$$

(7) $$\mathrm{BaO}+2{\mathrm{NO}}_{2}+\frac{1}{2}O_{2}\to \mathrm{Ba}{\left({\mathrm{NO}}_{3}\right)}_{2}$$

It is generally accepted that NO_2_ is the main species stored on a model NSR catalyst [1]. Fridell et al. [5] proposed that the adsorption of NO_2_ involves a pathway in which the adsorption of three molecules of NO_2_ implies the desorption of one molecule of NO, by following the general Equation (1). The NO_2_/O_2_ adsorption–desorption experiments under temperature programmed conditions (Figure S4 in Supplementary Information) revealed that NO is released at the NO_2_ adsorption temperatures. Thus, it seems that the disproportionation mechanism (Equation (1)) takes place for NO_2_ storage on the BaTi_0.8_Cu_0.2_O_3_ catalyst.

Below 350 °C, nitrites are the main NOx species identified by DRIFTS for the BaTi_0.8_Cu_0.2_O_3_. Similar results were obtained by Lietti et al. [11], who considered nitrite formation to be a dominant pathway in the NOx storage process at low temperature for model NSR catalysts under NO/O_2_. These authors concluded that, at low temperature, NO is partially oxidized by O_2_ in the noble metal–alkaline oxide border and, subsequently, stored forming nitrite and avoided overoxidation of NO to NO_2_. Hence, the so-called “nitrite route” implies good contact between oxidation and storage sites but also requires that oxidation sites acts as a supplier of oxygen to BaO, which promotes the NO to nitrites oxidation.

To determine the activity of the catalyst for NO storage, NO adsorption experiments without O_2_ in the reaction gas composition were also carried out. In Figure 1b, NO_2_ is not detected at any temperature under NO/N_2_. Under this condition, the detection of nitrites (Figure 2b) by DRIFTS reveals that NO must be adsorbed on BaTi_0.8_Cu_0.2_O_3_, predominantly on BaO sites of the catalyst. As O_2_ is absent in this experiment, the perovskite must supply the oxygen needed for the NO to nitrite oxidation step. The characterization results previously discussed, allow concluding that the introduction of copper into the perovskite structure leads to an increase in oxygen vacancies and oxygen surface groups in the catalyst structure. Thus, this surface-active oxygen, and also the presence of a reducible cation (copper), facilitate oxidation of NO to nitrite. This assumption requires a good contact between the adsorption sites and the oxidation sites, suggesting that BaO has to be located on the surface of the BaTi_0.8_Cu_0.2_O_3_ catalyst. These results allow concluding that NO is also adsorbed by forming nitrites according to Equation (8):

(8) $$\mathrm{BaO}+\mathrm{NO}+M-O^{*}\to \mathrm{Ba}({\mathrm{NO}}_{2})+M-□$$

where M–O* is an oxidation active site, which could be either an activated oxygen site on the perovskite surface formed by the adsorption of O_2_ at an oxygen vacancy or a copper active site (M=Cu) and, M−□ is an oxygen vacancy generated due to oxygen consumption.

Evidence for both “nitrite” and “nitrate” routes has been observed in cyclic NOx storage-regeneration experiments carried out by DRIFTS at 300, 350, 400 and 450 °C with the BaTi_0.8_Cu_0.2_O_3_ catalyst (Figure S2a–d in Supplementary Information). Under isothermal conditions, the dominant NOx storage route is also highly dependent on the temperature. At low temperature (300 °C), NOx is adsorbed as nitrite on the perovskite catalyst during the initial stages of the NOx storage cycle; however, these nitrites show a short lifetime, and after ~90 s, the bands corresponding to nitrites start to disappear, as these species are oxidized to nitrates by O_2_. Afterwards, only nitrates are detected as NOx stored species. By increasing the temperature, the lifetime of the nitrite species decreases and, at 450 °C, these species are no longer identified at the initial stages of the NOx storage indicating that the nitrite to nitrate oxidation step is promoted by increasing the temperature. As previously shown, the activity of catalyst for oxidation increases with temperature, thus favoring the fast oxidation of nitrites to nitrates, so, nitrites are not identified even at the beginning of the NOx storage cycle. Additionally, at high temperatures, NO_2_ generation is also improved, promoting the direct NO_2_ adsorption to form nitrates (nitrates route).

In summary, in situ DRIFTS experiments revealed that, for model NSR catalysts, NOx storage on BaTi_0.8_Cu_0.2_O_3_ catalyst takes place by both “nitrite” and “nitrate” routes, with the dominant pathway being highly dependent on the catalyst temperature and time on stream. At low temperatures (T < 350 °C), nitrites are formed on the catalyst, and are subsequently oxidized to nitrates. Above 350 °C, the high oxidation activity of the perovskite promotes the nitrite to nitrate oxidation and the NO_2_ generation and, consequently, the nitrate route prevails.

## 5. Conclusions

The incorporation of copper into the BaTi_0.8_Cu_0.2_O_3_ perovskite structure induces a segregation of predominantly BaCO_3_ and Ba_2_TiO_4_, but also CuO. XRD shows that Ba_2_TiO_4_ is an active phase for NOx storage, forming barium titanate and barium nitrate as final products. However, after nitrate decomposition, BaO is generated on the catalyst surface instead of Ba_2_TiO_4_. The estimation of the amounts of BaCO_3_ by ATR, TGA and XPS reveals that this BaO plays a key role during the NOx storage process on the BaTi_0.8_Cu_0.2_O_3_ catalyst.

Nitrites and nitrates are detected for the BaTi_0.8_Cu_0.2_O_3_ catalyst by in situ DRIFTS experiments under temperature programmed conditions and NOx storage–regeneration cycles at different temperatures. This fact indicates that, as for model NSR catalysts, both “nitrite” and “nitrate” routes take place during the NOx storage process.

A strong correlation between the oxidation activity of the perovskite and the NOx storage were observed. At low temperature (T < 350 °C) nitrites are formed on the catalyst by NO adsorption which are subsequently oxidized to nitrates, due to the participation of activated oxygen and copper (as redox active metal) on the perovskite surface. Above this temperature, the high oxidation activity shown by the catalyst enhances NO_2_ generation and the role of the nitrate route in the NOx storage mechanism. Additionally, this high oxidation activity promotes the nitrites to nitrates oxidation. Thus, as for model NSR catalysts, it can be concluded that the dominant pathway for NOx storage on BaTi_0.8_Cu_0.2_O_3_ catalyst is highly dependent on the catalyst temperature and on the time on stream.

## Figures and Tables

**Table 1:** NOx storage capacity (NSC) of the BaTi_0.8_Cu_0.2_O_3_ catalyst at different temperatures in 500 ppm NO + 5% O_2_ balanced with N_2_.

Temperature (°C)	NSC (µmol/g.cat.)	NO_2_/NOx (%)
300	211	5
350	231	11
400	262	16
450	340	15

**Table 2:** Estimation of the BaCO_3_ percentage by ATR, TGA, and XPS (atomic C percentage).

Catalyst	ATR (*w*/*w* %)	TGA (*w*/*w* %)	XPS (*w*/*w* % of C)
BTCuO_2_NSR	7.7	5.6	3.5
BTCuO_2_sat	4.9	4.9	1.9
BTCuO_2_red	6.5	5.8	2.7

## Supplementary Materials

The following are available online at https://www.mdpi.com/article/10.3390/nano11082133/s1, Figure S1: BaTi_0.8_Cu_0.2_O_3_ characterization data: (a) DRX diffractograms, and (b) Raman spectra, (c) XPS spectra corresponding to Cu2p3/2 and O1s transitions, and (d) H_2_-TPR profiles.; Figure S2: DRIFT spectra recorded for the BaTi_0.8_Cu_0.2_O_3_ catalyst during a NOx storage cycle at (a) 300 °C, (b) 350 °C, (c) 400 °C and (d) 450 °C in 500 ppm NO + 5% O_2_ in N_2_ atmosphere; Figure S3: XPS of the C1s transition for the BaTi_0.8_Cu_0.2_O_3_ catalyst after 5 NOx storage–reduction cycles (BTCuO_2_NSR), followed by 1 h term NOx adsorption experiment in 500 ppm NO + 5% O_2_ in N_2_ atmosphere (BTCuO_2_sat) and finally regenerated in 10% H_2_/N_2_ atmosphere; Figure S4: NO and NO_2_ concentration registered for BaTi_0.8_Cu_0.2_O_3_ catalyst during a TPR experiment in 500 ppm NO + 5% O_2_ atmosphere balanced with N_2_. (see details in Experimental Section); Figure S5: NO and NO_2_ concentration registered for BaTi_0.8_Cu_0.2_O_3_ catalyst during a TPR experiment in 500 ppm NO_2_ + 5% O_2_ atmosphere balanced with N_2_. (see details in Experimental Section); Table S1: XPS characterization data for BaTi_0.8_Cu_0.2_O_3_.
