# Supplementary material for: NOx Storage on BaTi0.8Cu0.2O3 Perovskite Catalysts: Addressing a Feasible Mechanism
Source: Nanomaterials (Basel). 2021 Aug 21;11(8):2133. doi: 10.3390/nano11082133 (PMC8401998; doi:10.3390/nano11082133)
Supplement: Supplementary file 1 [file nanomaterials-11-02133-s001.zip › nanomaterials-1319647-supplementary.pdf]

SUPPLEMENTARY INFORMATION

NOx storage on $BaTi_{0.8}Cu_{0.2}O_3$ perovskite catalysts: addressing a feasible mechanism.

Vicente Albaladejo-Fuentes[1], María-Salvadora Sánchez-Adsuar[1], J. A. Andersson[2], María-José Illán-Gómez[1,*]

[1]Carbon Materials and Environment Research Group, Department of Inorganic Chemistry, Faculty of Science, Universidad de Alicante, Alicante, Spain

[2] Surface Chemistry and Catalysis Group, School of Engineering, University of Aberdeen, AB24 3UE Scotland, UK

** Corresponding author: illan@ua.es. Phone: +34965903975.*

a)

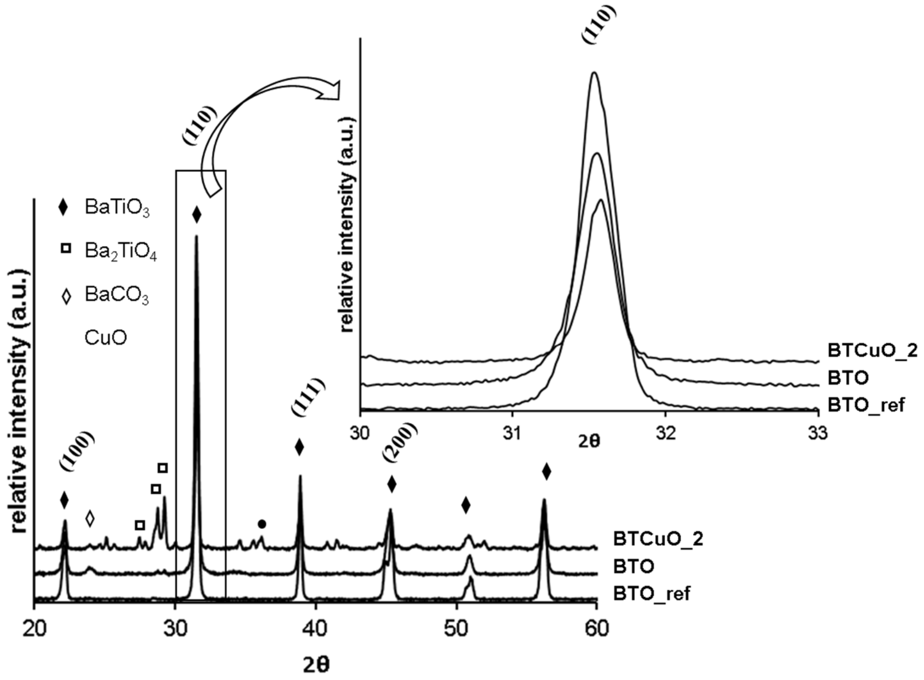

b)

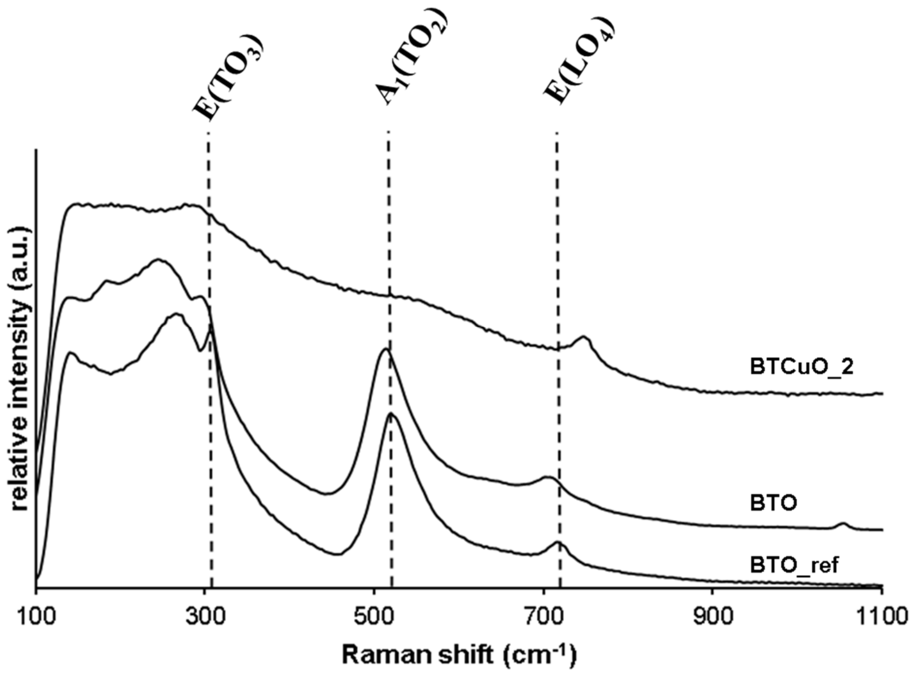

Figure.S1a-b. $BaTi_{0.8}Cu_{0.2}O_3$ characterization data: a) DRX diffractograms, and b) Raman spectra,

c)

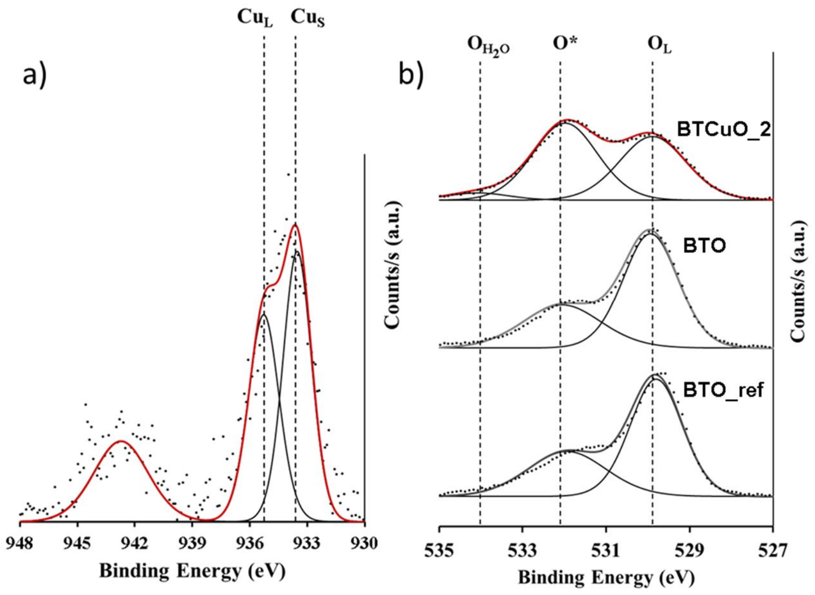

d)

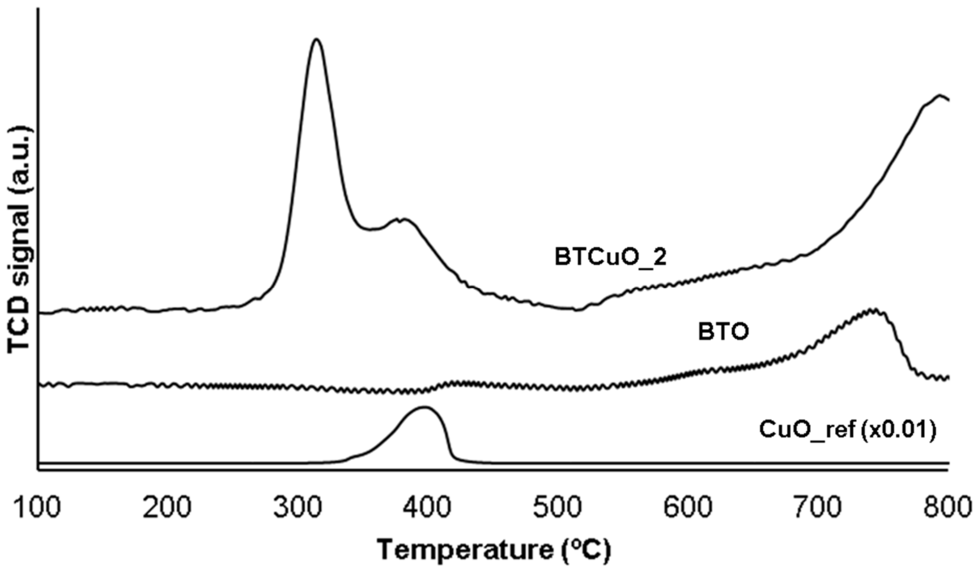

Figure.S1c-d. $BaTi_{0.8}Cu_{0.2}O_3$ characterization data: c) XPS spectra corresponding to Cu2p3/2 (a) and O1s (b) transitions , and d) H_2-TPR profiles.

Table S1. XPS characterization data for $BaTi_{0.8}Cu_{0.2}O_3$

Catalyst	Cu/ (Ba+Ti+Cu) (XPS)	Cu/ (Ba+Ti+Cu) (nominal)	(Cu*+Ti)/ Ba (XPS)	O_L/(Ba+Ti+Cu) (XPS)
BTO ref	-	-	0.98	2.05
BTO	-	-	0.72	1.78
BTCuO_2	0.06	0.1	0.49	1.50

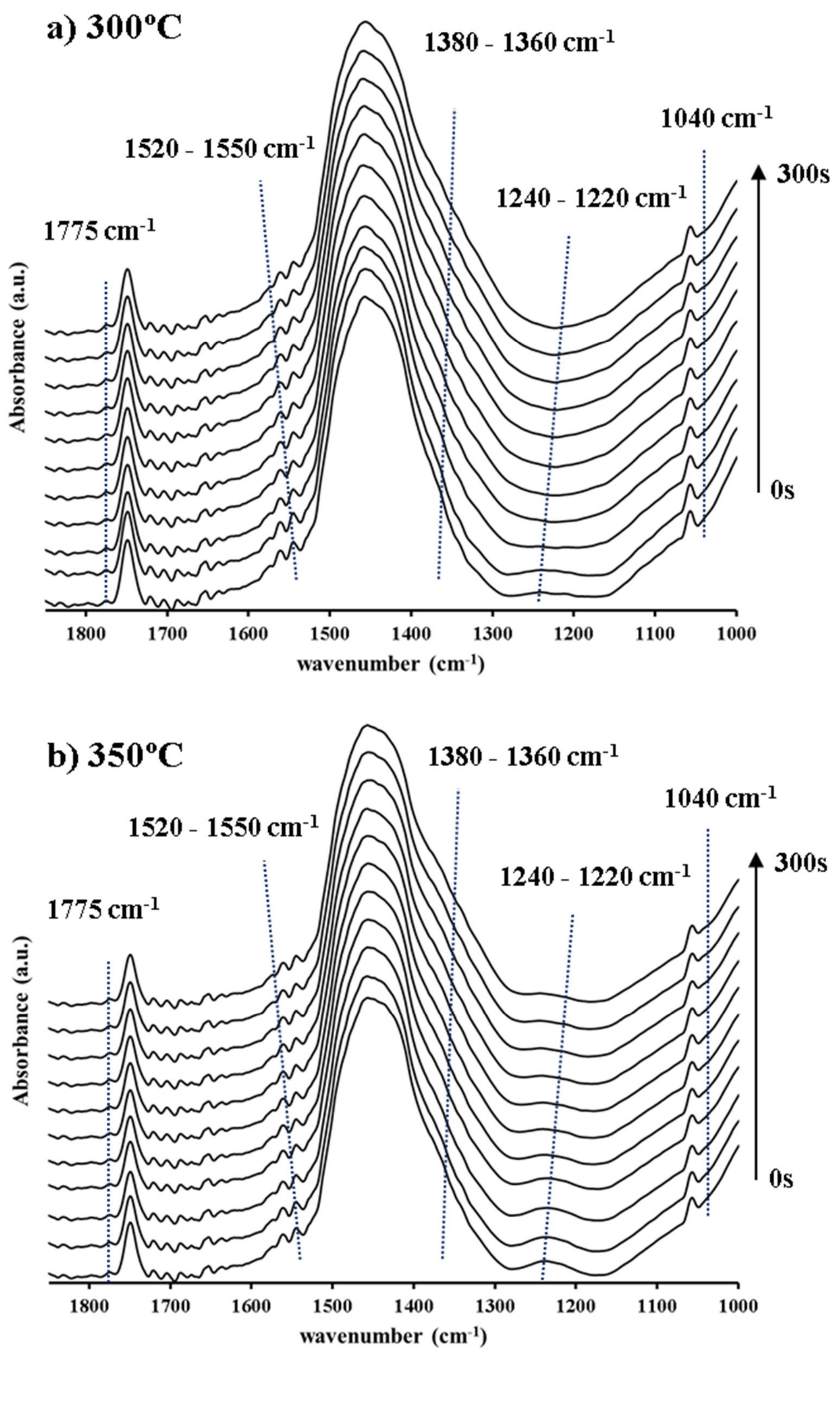

Figure S2a-b DRIFT spectra recorded for the $BaTi_{0.8}Cu_{0.2}O_3$ catalyst during a NOx storage cycle at a) 300ºC and d) 350ºC in 500 ppm NO + 5% O_2 in N_2 atmosphere.

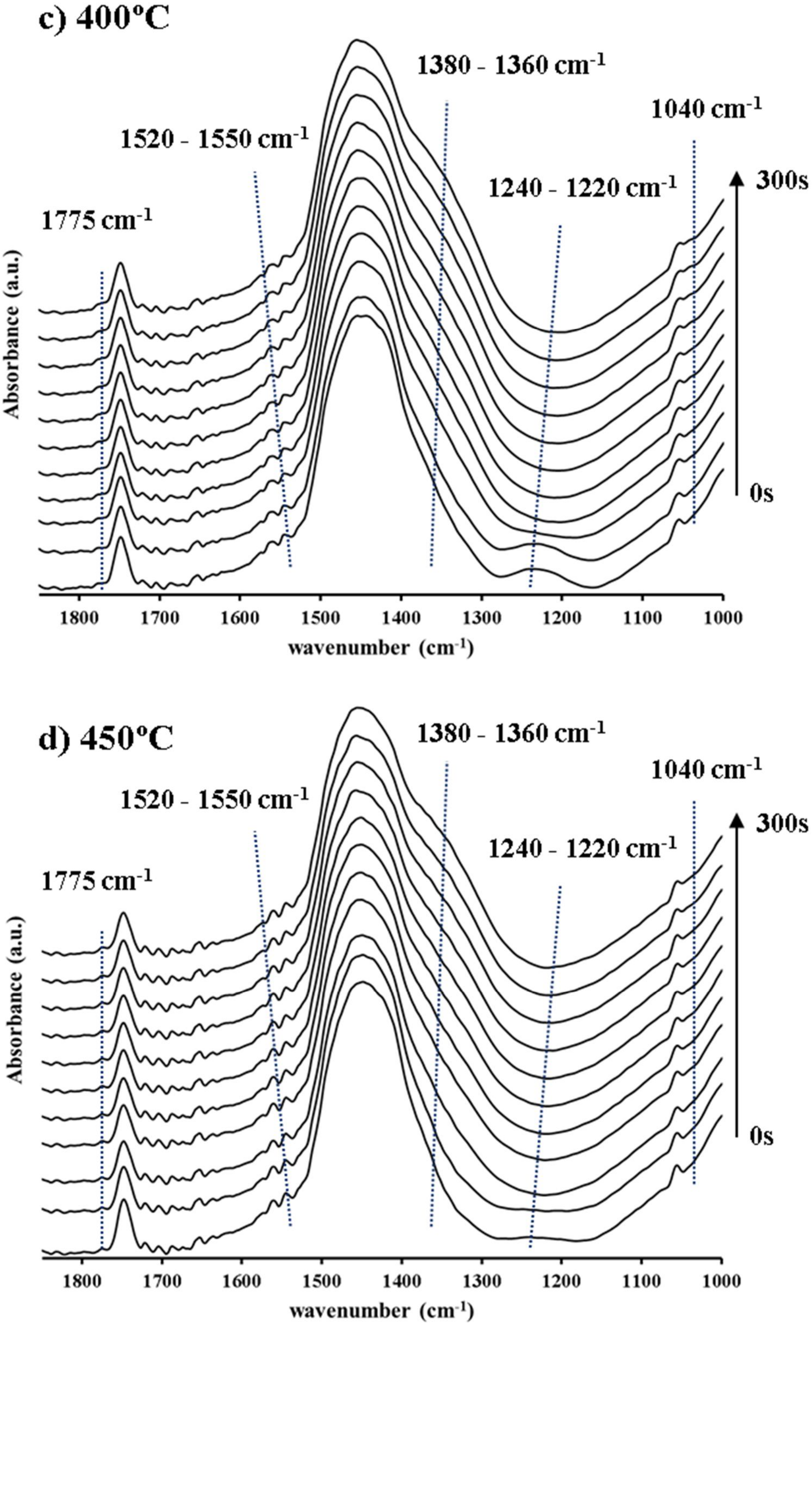

Figure S2c-d DRIFT spectra recorded for the $BaTi_{0.8}Cu_{0.2}O_3$ catalyst during a NOx storage cycle at a) 400ºC and d) 450ºC in 500 ppm NO + 5% O_2 in N_2 atmosphere.

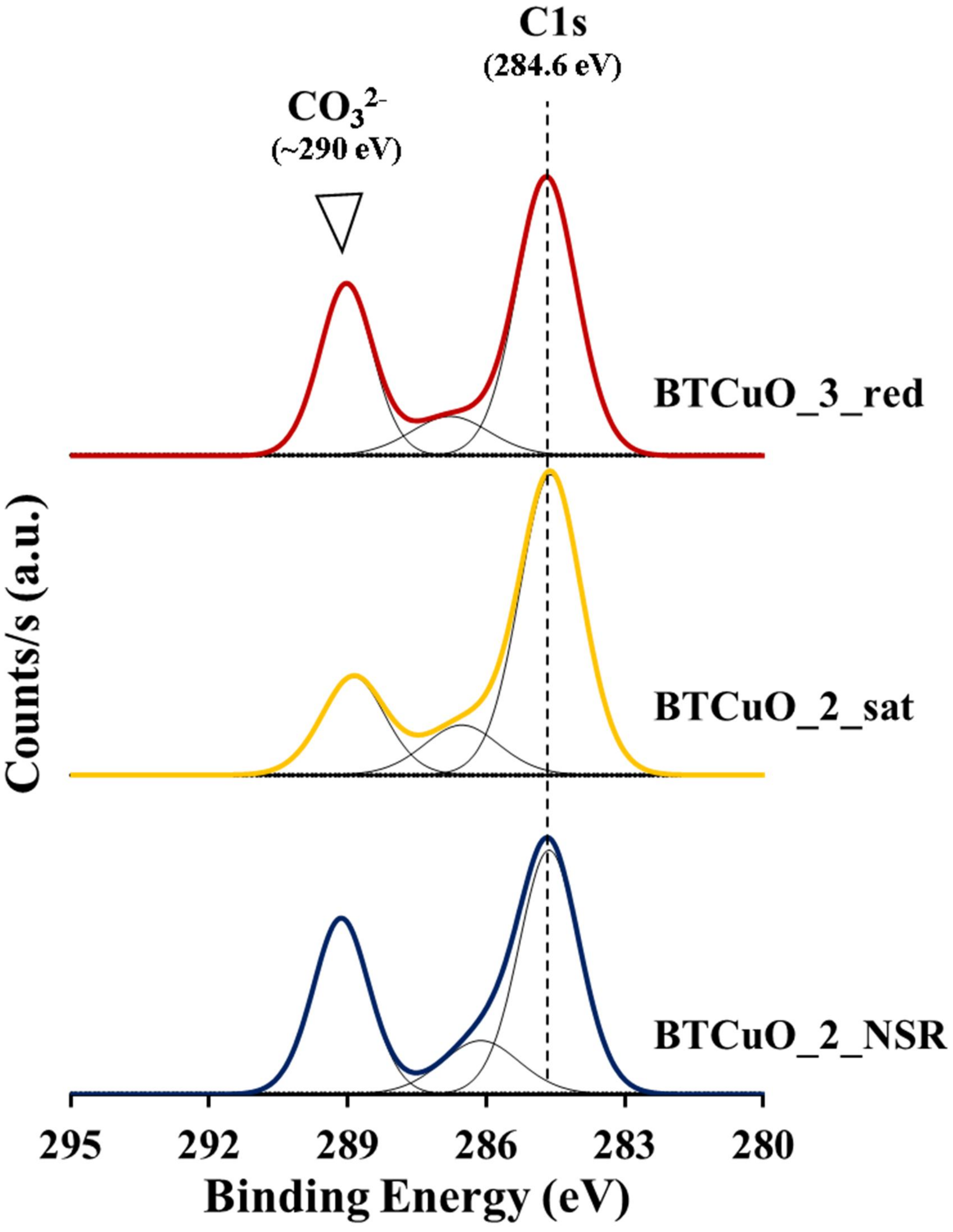

Figure S3 XPS of the C1s transition for the $BaTi_{0.8}Cu_{0.2}O_3$ catalyst after 5 NOx storage-reduction cycles (BTCuO_2_NSR), followed by 1 hour term NOx adsorption experiment in 500 ppm NO + 5% O_2 in N_2 atmosphere (BTCuO_2_sat) and finally regenerated in 10% H_2/N_2 atmosphere.

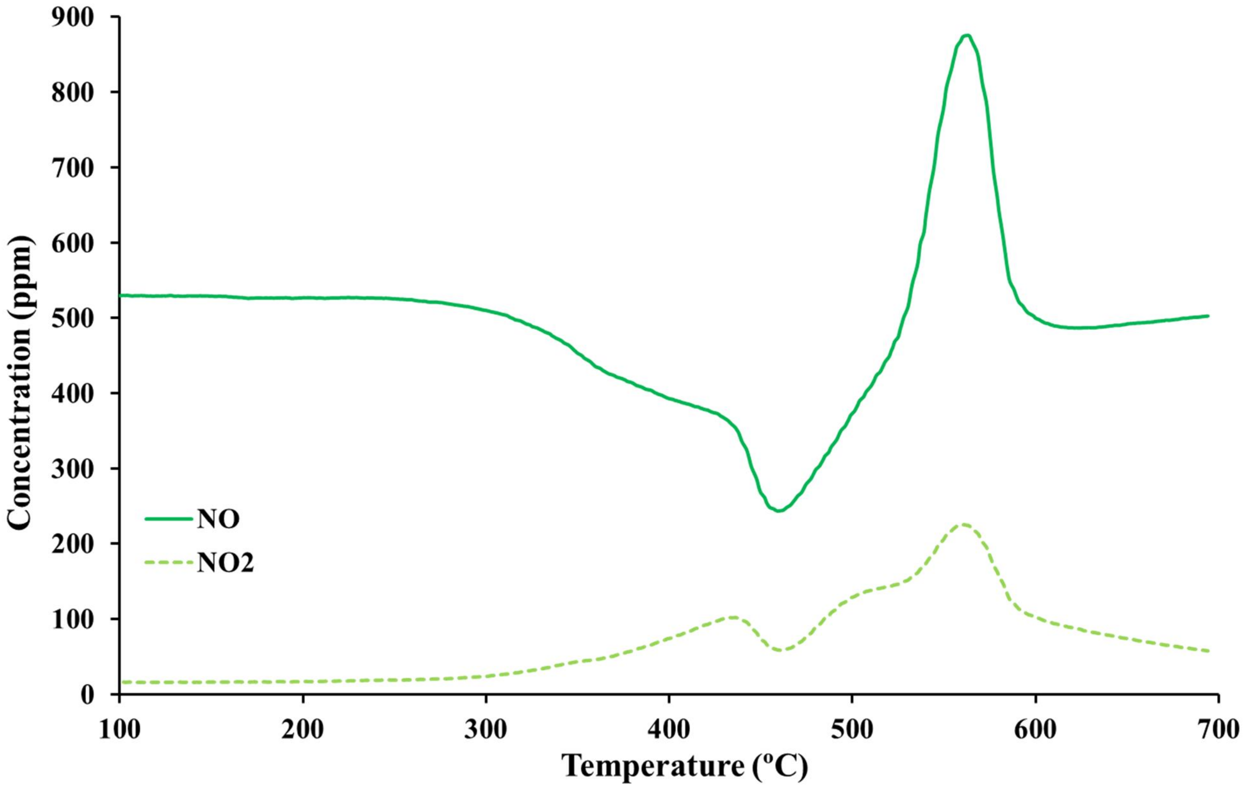

Figure S4 NO and NO_2 concentration registered for $BaTi_{0.8}Cu_{0.2}O_3$ catalyst during a TPR experiment in 500 ppm NO + 5% O_2 atmosphere balanced with N_2. (see details in Experimetal Section)

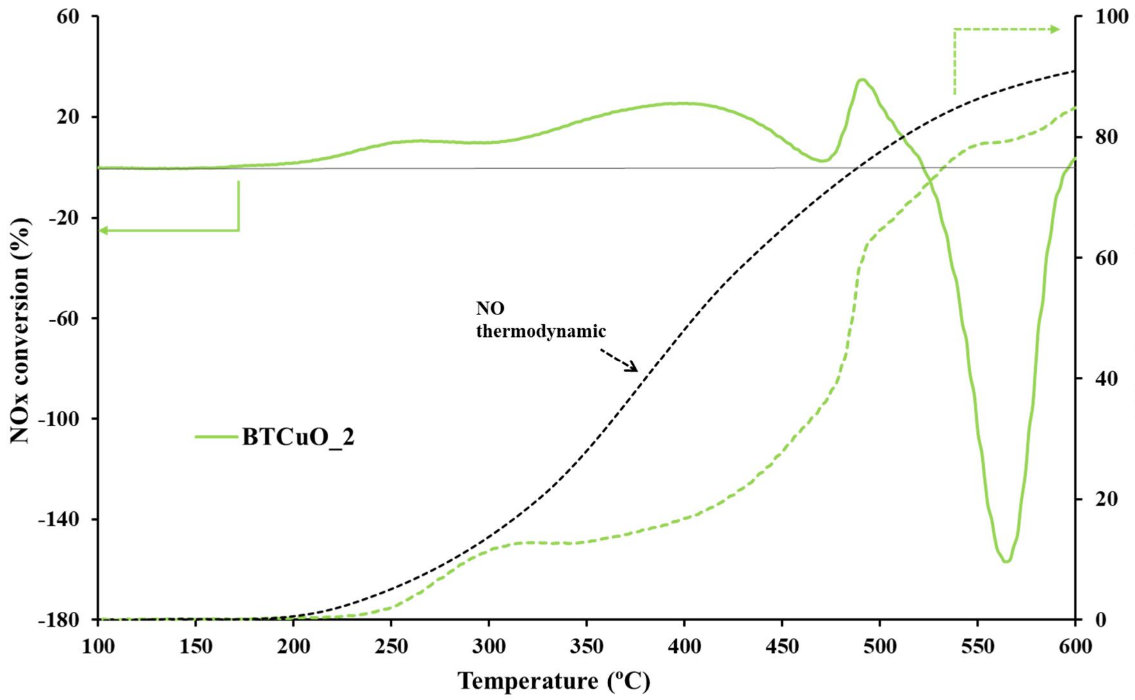

Figure S5 NO and NO_2 concentration registered for $BaTi_{0.8}Cu_{0.2}O_3$ catalyst during a TPR experiment in 500 ppm NO_2 + 5% O_2 atmosphere balanced with N_2. (see details in Experimental Section).
